# Spatiotemporal dynamics of terrestrial invertebrate assemblages in the riparian zone of the Wewe river, Ashanti region, Ghana

**DOI:** 10.1515/biol-2020-0037

**Published:** 2020-06-13

**Authors:** Collins Ayine Nsor, Samuel K. Oppong, Emmanuel Danquah, Michael Ochem, Osei Owusu Antobre

**Affiliations:** Department of Forest Resources Technology, Faculty of Renewable Natural Resources, Kwame University of Science and Technology, Kumasi, Ghana; Department of Wildlife and Range Management, Faculty of Renewable Natural Resources, Kwame University of Science and Technology, Kumasi, Ghana

**Keywords:** habitat conditions, environmental disturbance, geometric series, rarefaction, Renyi diversity ordering, canonical correspondence analysis

## Abstract

This study assessed invertebrate response to disturbances in the riparian zone of the Wewe river, using geometric series, rarefaction, Renyi diversity, and CCA models. We sampled 2,077 individuals (dry season) and 2,282 (wet season) belonging to 16 invertebrate orders. The severely disturbed habitat registered the highest individuals (*n* = 1,999), while the least was the moderately disturbed habitat (*n* = 740). Seasonal assemblages were not significantly different. Fire, farming, tree felling, and erosion explained 66.8% and 60.55% in the dry and wet seasons, respectively, of variations in invertebrate assemblages. This suggests threats to the invertebrate community and the riparian ecosystem health by anthropogenic interventions.

## 1 Introduction

Riparian zones, as the transition between terrestrial and aquatic systems, serve as refugia for most invertebrates and play a critical role in ecosystem functioning and human lives [1,2,3]. Many of their roles in the ecosystem include detection of habitat degradation, decomposition of organic materials, and nutrient cycling, as biological indicators of pollution and ensuring continuity in ecological food chains [2,3]. The differing roles of terrestrial invertebrates in the natural ecosystem are part of the processes that keep the ecosystem in equilibrium *viz-a-viz* the spread of diseases, checking the population of organisms, and elimination of alien invasive species and source of food for other animals and plants [4].

Ecological disturbances within the catchment of riverine systems in urban centers have often led to transformation or loss of riparian vegetation [5,6]. Being a refugium for many invertebrates [7], fragmentation of riparian zones, following disturbances such as farming practices, grazing, and logging, could potentially reduce their habitats into narrow ranges and consequently affect community structure. Ecologists have used terrestrial invertebrates as indicators of ecological condition or stream biological integrity due to their high sensitivity to disturbances and wide distribution [8,9,10]. A study has revealed about 45% reduction in global terrestrial invertebrate population in the current Anthropocene epoch, which could likely cascade onto ecosystem functioning and human well-being [11]. What is particularly worrying is the rapid decline in a number of insect pollinators and a shift in their community ranges, which could soon translate into less frequent flower visitation and gradual reduction of seed and fruit production [12]. The cause of this population decline and the altering of their composition have been linked largely to human activities such as farming, logging, grazing, burning, and urbanization along rivers and streams [6,13,14]. Due to these human-led disturbances, terrestrial macroinvertebrates have attracted conservation concerns and are broadly considered as targets of conservation efforts in many countries [15,16,17,18]. Research on the community assemblages of terrestrial macroinvertebrates (i.e., a combination of adult stages of aquatic invertebrates and semi-aquatic taxa) and how they are influenced by environmental variables are therefore crucial, considering that the aim of biodiversity monitoring is to track changes in the biological integrity of ecosystems [19].

In Ghana, most rivers in urban centers such as the Wewe river are undergoing rapid degradation due to agricultural expansion and infrastructural development [20,21,22]. These emerging threats in the riparian zones of the Wewe river tend to impact on macroinvertebrate assemblages, their habitats, and overall hydrologic processes and connectivity. Studies on the response of terrestrial invertebrate communities to disturbances among urban riparian zones of Ghana are scant (e.g., [23]). The Wewe river is one of the few systems in the Kumasi Metropolitan Area that drains through a forest reserve and serves as a major source of groundwater recharge, supporting the riparian vegetation and other nearby aquifers. Terrestrial macroinvertebrates that inhabit these riparian zones [24] play a crucial mediated role as pollinators [12], leaf litter decomposition [25], and soil aerators of urban soil microbiome [26]. However, in recent times, there have been worrying concerns about the increasing level of ecological disturbances along the Wewe riparian zone, namely, farming, sewage disposal, tree felling, and bushfire [27]. It is unclear how these environmental drivers have affected the macroinvertebrate assemblages, which play a key role in ecosystem functioning [2,3]. Thus, given their important ecological role of macroinvertebrates as pollinators [12], soil nutrient cycling/soil aerators [26], and a source of food for other riparian animals, understanding how prevailing environmental drivers have impacted on macroinvertebrate assemblages in the riparian zone of the Wewe river is critical in determining the right conservation measures to implement, to protect them and improve on their habitat quality. The purpose of this study is to establish a baseline information on riparian macroinvertebrate assemblages in an urban environment such as the Kumasi Metropolitan Area of Ghana and to develop a sensitive suit of indicator species used as a monitoring tool for riparian habitat quality, through the assessment of species threshold tolerance to ecological disturbances between seasons. We hypothesized that: (a) invertebrate abundance, taxon richness, and diversity will not differ between seasons, because the survival of some invertebrates is not seasonally dependent [28]; and (b) processes such as farming, sewage disposal, tree felling, creation of bare ground and bushfire that influence invertebrate communities will vary in the wet and dry seasons, giving that intensity and scale of disturbances are sometimes influenced by seasonal variations, which reflect in deferring responses by the biotic components [27].

## 2 Materials and methods

### 2.1 Study area

**Figure 1:**
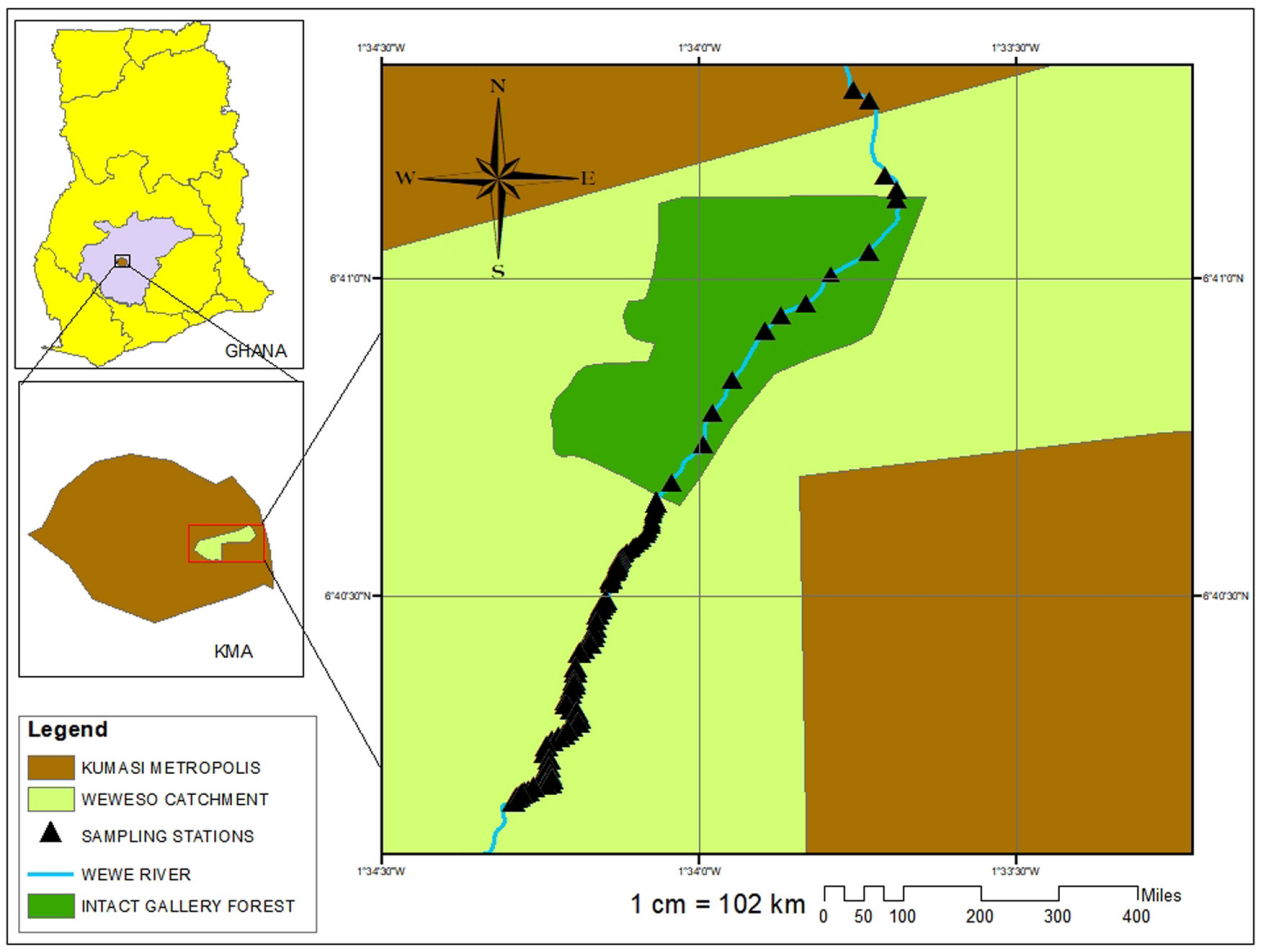
Map of Ghana showing the study area in the Kumasi Metropolitan Area (Ashanti region).

**Figure 2:**
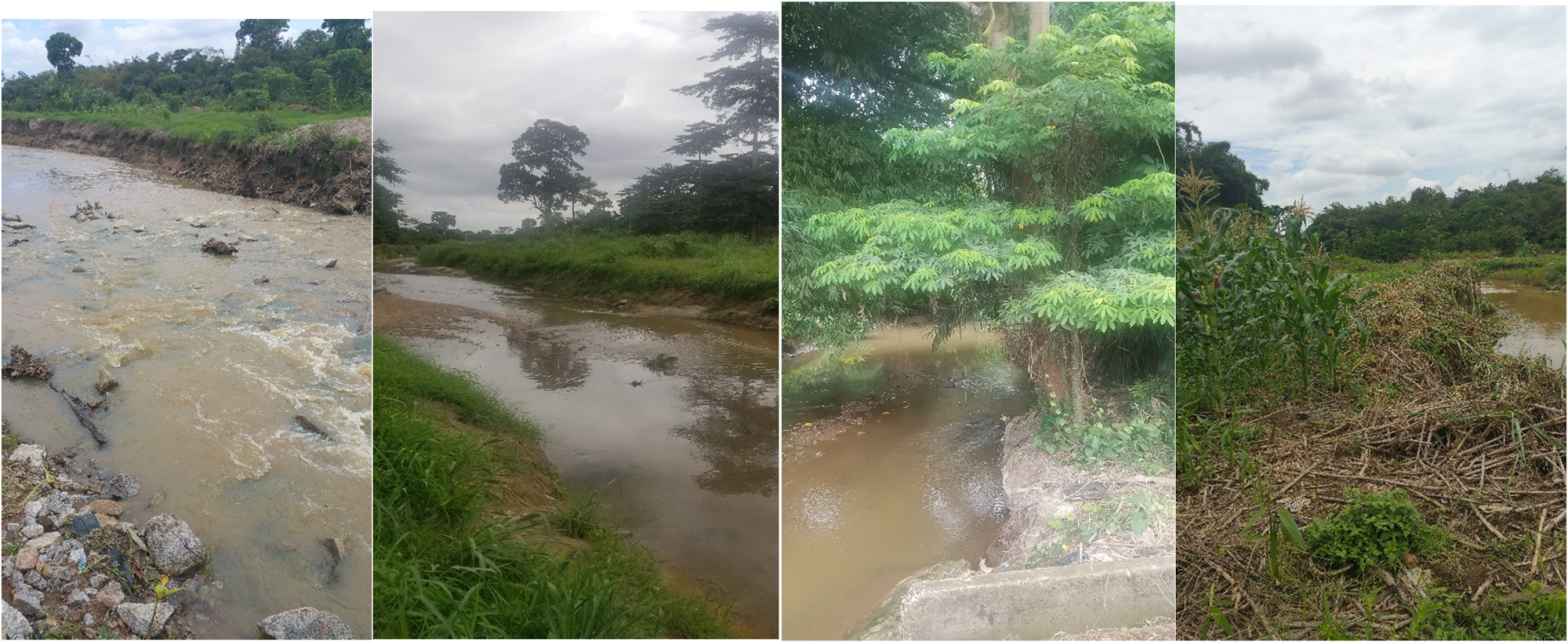
Photographs of some segments of the riparian habitats along the Wewe river, where the study was conducted.

The Wewe river is among the many drainage systems in the Kumasi Metropolitan Area of Ghana and located within N 06° 41′ 301″ W 001° 33′ 744″ and N 06 ° 40′ 329″ W 001° 34′ 20.9″ (Figures 1 and 2). The study area falls within one of the urban forest reserves, with a substantial number of economic tree species [29]. In recent times, human-led disturbances, namely, farming, burning, grazing, and infrastructure development, have led to a significant transformation of the riparian zone. Soils are typically heavy clay to loamy, characterized by cobbles and boulders. The rock type is igneous and metamorphic rocks, with undulating topography. The average temperature is 24–34°C p.a. and generally humid. The rainfall pattern is typically bimodal, with an annual average of 2,000 mm [30].

### 2.2 Classification of the sampling sites on the riparian zone

Before conducting the actual study, we embarked on a ground-truthing reconnaissance survey to demarcate the riparian zone, based on the habitat condition, and this included: intact, moderate, and severe habitat conditions, following Riparian Quality Index (RQI) methods [31], which represents a useful tool for monitoring and evaluating the structure of riparian zones, an element of the river morphological conditions (i.e., gravel zones, sand dunes, boulders, and bare ground) considered by the Water Framework Directive [31]. The index ranges from 1 to 15. Thus, intact condition class (10–15): areas dominated by different vegetation strata that cover the full length of the segment, which is linked to natural fluvial forms and slightly fragmented; moderately disturbed condition class (7–9): areas with vegetation cover nearly half of the study zone being disturbed, 1–3 m active channel width and about 10–30% exotic and ruderal species present. Severely disturbed condition class (1–6): areas where 60% of the riparian corridor is reduced by human-led activities, vegetation covering <30% (mainly grasses/herbs and isolated woody species) with channel banks connected to agricultural fields. Based on these habitat conditions, the upstream, midstream, and downstream of the riparian zone were classified as moderately disturbed habitat, intact habitat, and severely disturbed habitat, respectively.

### 2.3 Sampling procedure for macroinvertebrates

Invertebrates were collected randomly in 145 sample stations (i.e., 5 m radius per sample station) in all the three habitats along the riparian zone of the Wewe river. Invertebrates were then sampled within each of the 5 m radii, using a wooden-handle sweep net of length 85 cm, aperture 30 cm, net length 60 cm, and mesh size of 0.5 × 0.75 mm from knot-to-knot [32,33]. Sweep netting is one of the methods for sampling terrestrial invertebrates over a large area and is advantageous for sampling remote wetlands [2,34]. Repeated sweeps of ∼10× per 5 m radius were undertaken, to increase the rate of catchability or detectability. A total of 8 hours of sampling session per day [34] was undertaken, beginning at 08:00 GMT where most invertebrates were noticeably present and active, and over a 7-month period (3 months in the dry season and 4 months in the wet season [35,36]. Sampled invertebrates were quickly transferred into a well-covered Petri-dish to prevent them from escaping and labeled according to the sample station and site that they were collected. Invertebrates were subsequently pinned to reduce mobility during identification in the laboratory, using Field Guide to Insects of South Africa, provided by [37].

### 2.4 Environmental assessment

Environmental drivers, namely, farming activities, sewage disposal, tree felling, bare ground, and bushfire, were measured based on the severity and scope of their threats on invertebrate assemblages, using the Battisti et al. [38] model approach. These environmental drivers were assessed to determine how invertebrates responded to the threats. A score ranging from 1 to 4 (i.e., 1 = lesser impact and 4 = highest impact) was used to assess the scope and severity of identified threats. For “scope”, we referred to the percentage ratio of the sample plot affected by a specific threat within the last 5 years [38] in each habitat. Here, a one-on-one field interview with some users of the Wewe river was undertaken to determine whether each of the identified threats persisted in the last 5 years. The score for each identified threat per plot was then averaged for all plots in each habitat, to determine the overall score for all threats. The scores were assigned as follows: 4: the threat is found throughout (50%) the sample station; 3: the threat is spread in 15–50% of the sample station; 2: the threat is scattered (5–15%); and 1: the threat is localized (<5%). Identifying how many and the types of threats present and their regime [39] is critical when assessing the invertebrate community structure, particularly in a disturbed ecosystem like the Wewe river, for effective management.

### 2.5 Statistical analysis

Invertebrate abundance as a measure of diversity was quantified using a rank abundance model [40]. In each of the three habitat condition classes, we listed the number of invertebrate orders say *S*1 represented by one individual and the number of orders, say *SK*, represented by *K* individuals, where *K* denotes the abundance of the most abundant order and *S1* +,…,+ *SK* = *S* [41]. Accordingly, the sequence of relative frequencies *fr* = *Sr*/*S* (*r* = 1,…,*K*) represents a frequency distribution for the number of individuals per species which is often referred to as the species–abundance curve [41]. Geometric series (GS) was then fitted to the invertebrate data (raw abundance) using the regression model approach [42], to evaluate how the orders were assembled in each of the habitats. We used the GS model because we sought to test against the null hypothesis (*H*_0_) that invertebrate order abundance distribution and richness did not differ across the three habitat types. All the insect orders per habitat were ranked from the most to the least abundance on the rank abundant curve [43]. Each insect order rank was plotted on the *x*-axis and the abundance plotted on the *y*-axis. With the geometric series, if a log scale is used for abundance, the species exactly fall along a straight line, according to the model equation

(1) \log \hspace{.25em}A=\hspace{.25em}{b}_{\text{1}}+\hspace{.25em}{b}_{1}R

where *A* is the species abundance, *R* is the respective rank, and *b*_0_ and *b*_1_ are optimized fitting parameters [43]. Geometric series was preferred over the log-series because it facilitates a better comparison of invertebrate order abundance distribution among habitat types [42].

An individual-based rarefaction technique was used to compare insect order richness across the three habitats (rarefaction curves) [44]. Rarefaction curves are created by randomly re-sampling the pool of *N* samples a number of times and then plotting the average number of orders found in each sample (1, 2,…,*N*) [45]. This generates the expected number of orders in a small collection of *n* individuals or *n* samples drawn at random from the large pool of *N* samples. The rarefaction curve *f*_*n*_ is defined as

(2) {f}_{n}=E\left[{X}_{n}\right]=K-{\left(\begin{array}{c}N\\ n\end{array}\right)}^{-1}\mathop{\sum }\limits_{i=1}^{k}\left(\begin{array}{c}N-{N}_{i}\\ n\end{array}\right)

where *X*_*n*_ is the number of groups still present in the subsample of “*n*” less than *K* whenever at least one group is missing from this subsample, *N* is the total number of items, *K* is the total number of groups, *N*_*i*_ is the total number of items in group *i* (*i* = 1,…,*k*) [45,46]. Rarefaction methods (both sample and individual-based) allow for a suitable standardization and comparison of invertebrate datasets with different sampling effort [47,48].

The Shannon entropy model or Renyi diversity ordering [49,50] was used to quantify insect order diversity among the three habitats. This model has the ability to bring together the different diversity indices used for biodiversity analysis (e.g., Berger–Parker, Shannon–Weiner, Simpson’s 1_D, diversity, Pielou evenness indices), which hitherto made it difficult to select the appropriate model index for comparing biodiversity measurements [40]. Renyi [49] extended the concept of Shannon’s entropy [51], by defining the entropy of order alpha (*α*) as

(3) (\alpha \ge 0,\alpha \ne 1)\hspace{.5em}\text{of}\hspace{.5em}\text{a}\hspace{.5em}\text{probability}\hspace{.5em}\text{distribution}\hspace{.5em}\text{(}{p}_{\text{1}},{p}_{\text{2}}\ldots {p}_{\text{s}}\text{)}

Diversity profile values (*H*-alpha) were calculated from the frequencies of each component species (proportional abundances *p*_*i*_ = abundance of species *i*/total abundance) and a scale parameter (*α*) ranging from zero to infinity as [52]

(4) ({H}_{\alpha })=\left(\log \hspace{.25em}\mathop{\sum }\limits_{i-1}^{s}{{p}_{i}}^{\alpha }\text{ }\right)/1-\alpha

Canonical correspondence analysis (CCA) [53] was performed to determine the relationship between insect order distribution and environmental stressors. CCA is a direct ordination method, with the resulting product being the variability of the environmental data, as well as the variability of species data [54]. To remove multicollinearity (i.e., perfect correlation with other predictive factors, which tend to inflate variances of the parameter estimates), we performed the ridge regression method [55,56]. This variant of the least squares regression model approach ensures a smaller variance in the resulting parameter estimates, by initially examining the variance inflation factor (VIF) and tolerance [56]. Kruskal–Wallis test (a non-parametric technique) was used to test for a significant difference in insect order abundance distribution, taxon richness, and diversity, since the initial test showed that plots were not normally distributed (*W* = 0.86, *p* = 0.92, Shapiro–Wilk test). Student *t*-test was performed to determine the seasonal difference among insect orders, whereas a one-way ANOVA test was used to determine whether environmental factors differed within and between the dry and wet seasons. Where significant difference was detected, we further employed the Tukey HSD *post hoc* test, to determine which habitats differed. A Spearman rank correlation test was performed to evaluate the significant relationship among environmental factors. All analyses were performed using PAST ver. 3.18 Package [57].


**Ethical approval:** The conducted research is not related to either human or animal use.

## 3 Results

### 3.1 Seasonal variations in invertebrate composition and abundance distribution pattern

**Figure 3:**
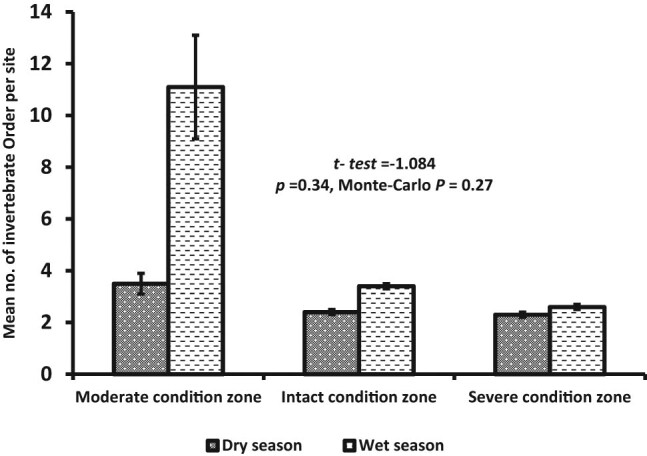
Changes in the seasonal composition of terrestrial macroinvertebrate across the three habitats of the riparian zone. Notice that macroinvertebrates were generally higher in the wet season than the dry season.

**Figure 4:**
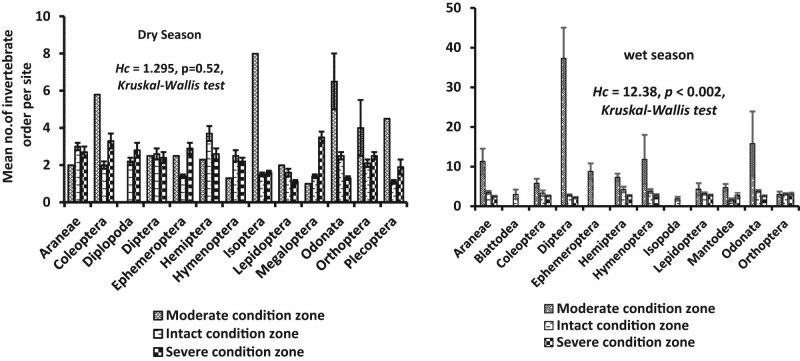
Mean composition of insect order in the three habitat condition zones of the Wewe river in the dry season. Notice that Isoptera and Diptera were the most dominant insect order, in the moderately disturbed habitat in the dry and wet seasons, respectively.

**Figure 5:**
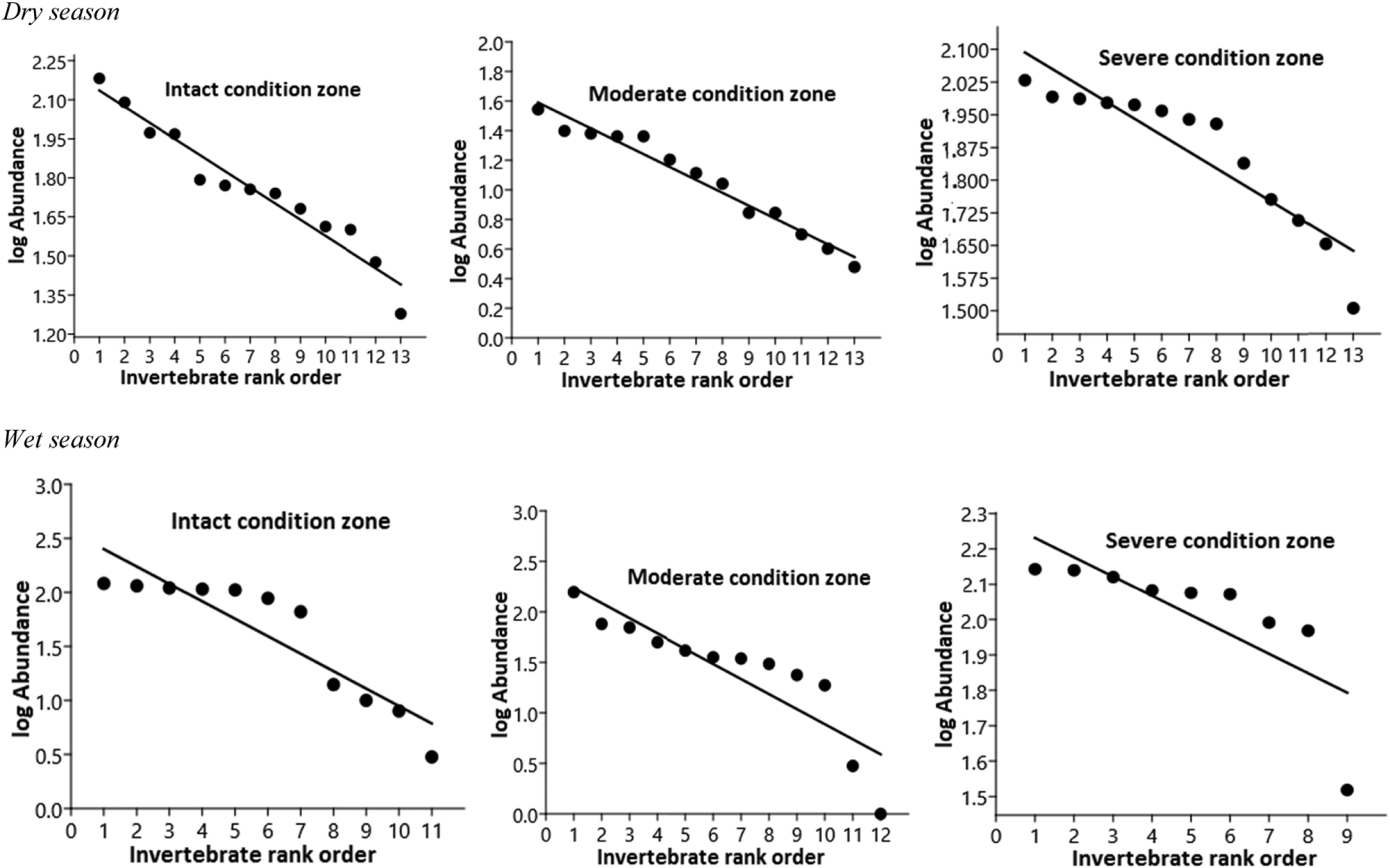
Geometric model for order rank abundance distribution across the three habitats in each season in the riparian zone. Abundance is based on cumulative count values per sampling site. Notice that SADs are ordered in decreasing magnitude and plotted against their corresponding rank.

A total of 4,359 individuals belonging to 16 insect orders were identified in the dry (*n* = 2,077) and wet (*n* = 2,282) seasons across the three habitats (Table 1). The severely disturbed habitat registered the highest number of individuals in the dry (*n* = 1,008) and wet (*n* = 991) seasons, followed by the intact habitat (dry = 960 and wet = 868 seasons) (Table 1). Seasonal abundance generally showed no significant difference (*t*-test = −1.084, *p* = 0.34), although mean abundance in the wet season (11.1 ± SE 2.0–2.6 ± 0.1) was marginally higher than the dry season (3.5 ± SE 0.4–2.3 ± 0.1) (Figure 3). Variations among individuals did not differ substantially among the three habitats in the dry (*H*_c_ = 1.295, *p* < 0.52, Kruskal–Wallis test), when compared with the wet season where we observed a significant difference (*H*_c_ = 12.38, *p* < 0.002, Kruskal–Wallis test) (Figure 4). Mean abundance among insect orders showed isoptera (8.0 ± SE 1.0) and diptera (37.3 ± SE 7.7) to be the highest in the dry and wet seasons, respectively, and which reflects their seasonal-specific preferences to habitat conditions only in the moderately disturbed habitat zone (Figures 4 and 5). Plecoptera (1.1 ± SE 0.1) and mantodea (1.6 ± SE 0.4) were the least detected in the dry and wet seasons, respectively, and found in the intact habitat. These two insect orders constituted 2.2% and 0.4%, respectively (Figure 4).

**Table 1:** Terrestrial invertebrate order and their percentage abundance among the three habitats in the riparian zone in the dry and wet seasons

Invertebrate order	Intact zone	Rel. abundance (%)	Moderate zone	Rel. abundance (%)	Severe zone	Rel. abundance (%)
*Dry season*						
Araneae	123	14.09	7	3.57	107	10.62
Coleoptera	62	7.10	35	17.86	97	9.62
Diplopoda	57	6.53	5	2.55	91	9.03
Diptera	93	10.65	16	8.16	85	8.43
Ephemeroptera	40	4.58	7	3.57	95	9.43
Hemiptera	152	17.41	23	11.74	94	9.33
Hymenoptera	55	6.30	13	6.63	98	9.72
Isoptera	48	5.49	24	12.25	51	5.06
Lepidoptera	30	3.44	4	2.04	45	4.46
Megaloptera	41	4.69	3	1.53	87	8.63
Odonata	94	10.77	25	12.76	57	5.66
Orthoptera	59	6.76	23	11.75	69	6.85
Plecoptera	19	2.17	11	5.61	32	3.18

Totals	873		196		1008	

*Wet season*						
Araneae	110	14.73	36	6.62	119	12.01
Blattodea	8	1.07	1	0.18	0	0
Coleoptera	14	1.87	42	7.72	132	13.32
Diptera	107	14.32	157	28.86	93	9.38
Ephemeroptera	0	0	35	6.43	0	0
Hemiptera	88	11.78	76	13.97	138	13.93
Hymenoptera	121	16.19	50	9.192	121	12.21
Isopoda	10	1.34	3	0.55	0	0
Lepidoptera	105	14.06	31	5.69	139	14.03
Mantodea	3	0.40	19	3.49	33	3.33
Odonata	115	15.39	70	12.87	118	11.91
Orthoptera	66	8.84	24	4.41	98	9.89
						
Totals	747		544		991	

Insect order abundance distribution fitted well in the geometric series distribution (GS) model and showed significant difference in the wet season (*F*-test = 8.703, *p*(regr) = 0.008, ANCOVA interactions × insect order rank) compared with the dry season (*F*-test = 2.755, *p*(regr) = 0.111, ANCOVA interactions × insect order rank) among the three habitats (Figure 5 and Table 2). However, from individual habitats, we observed a significant variation in insect order abundance along the slopes of the OAD curve in the moderately disturbed habitat (slope [*k*] = 0.465 ± 0.216, *R* ^2^ = 0.317, *χ* ^2^ *P* = 0.0009), intact (*k* = 0.682 ± 0.317, *R* ^2^ = 0.317, *χ* ^2^ *P* = 0.0011), and severely disturbed habitats (*k* = 0.599 ± 0.388, *R* ^2^ = 0.193, *χ* ^2^ *P* = 0.0017) in the wet season (Figure 5 and Table 2). The dry season tended to show no significant variations in their distribution patterns on the slope of the OAD curves for moderate (*k* = 0.082 ± 0.076, *R* ^2^ = 0.097, *χ* ^2^ *P* = 4.475), intact (*k* = 1.181 ± 1.085, *R* ^2^ = 0.097, *χ* ^2^ *P* = 4.933), and severely disturbed habitats (*k* = −0.089 ± 0.716, *R* ^2^ = 0.0014, *χ* ^2^ *P* = 1.387). Comparison of the variations in insect order abundance distribution among the three habitats facilitates the distinguishing of each habitat quality in relation to its influence on insect order success at competing for resources and adaptation to disturbances within their niche space. Thus, the least abundance of insect orders registered in the moderately disturbed habitat (dry = 109, and wet = 423 seasons) was more evenly distributed as shown in the shallow rank abundance curve, while severely disturbed habitat with the highest insect order abundance (dry = 1,008 and wet = 991 seasons) was less evenly distributed, as indicated in the steep rank abundance distribution curve (Figure 5 and Table 2).

**Table 2:** Results of the geometric series model for the abundance rank distribution of terrestrial invertebrate, calculated for all three habitats in each season

Samples	Intercept ± S.E.	Slope ± S.E.	*R* ^2^	Prob.
*Dry season*				
Moderate	9.548 ± 5.791	0.082 ± 0.076	0.097	4.475
Intact	49.353 ± 19.449	1.181 ± 1.085	0.097	4.933
Severe	78.877 ± 12.83	−0.089 ± 0.716	0.0014	1.387
Slope of OAD: *F*-test = 2.755, *p*(regr): 0.111				

*Wet season*				
Moderate	16.398 ± 17.043	0.465 ± 0.216	0.317	0.0009
Intact	31.324 ± 19.174	0.682 ± 0.317	0.317	0.0011
Severe	55.44 ± 23.474	0.599 ± 0.388	0.193	0.0017
Slope of OAD: *F*-test = 8.703, *p*(regr): 0.008				

Out of the 16 insect orders detected, five were found in all the three habitat condition zones during the wet season and constituted 65.38% of the total sampled (*n* = 2,282). They included Diptera (*n* = 357, log-rank abundance = 2.08), Odonata (*n* = 304, log-rank = 2.06), Hymenoptera (*n* = 292, log-rank = 2.04), Lepidoptera (*n* = 275, log-rank = 2.02), and Araneae (*n* = 264, log-rank = 2.02). Similar number of insect orders (Araneae = 237, Hemiptera = 269, Coleoptera = 194, Diptera = 194 and Odonata = 176) were detected in all three habitats during the dry season and represented 51.52% of the total sampled (*n* = 2,077). The presence of these five insect orders across the three habitats was indicative of their broad range habitat preference and tolerance to different disturbance regimes. Rarer insect orders such as Blattodea (*n* = 9, log-rank = 0.47) and Isopoda (*n* = 13, log-rank = 0.90) were the least ranked on the OAD curve and only occurred in the intact habitat during the wet season (Figure 5 and Table 2).

### 3.2 Invertebrate order richness and diversity along with the Wewe riverine system

**Figure 6:**
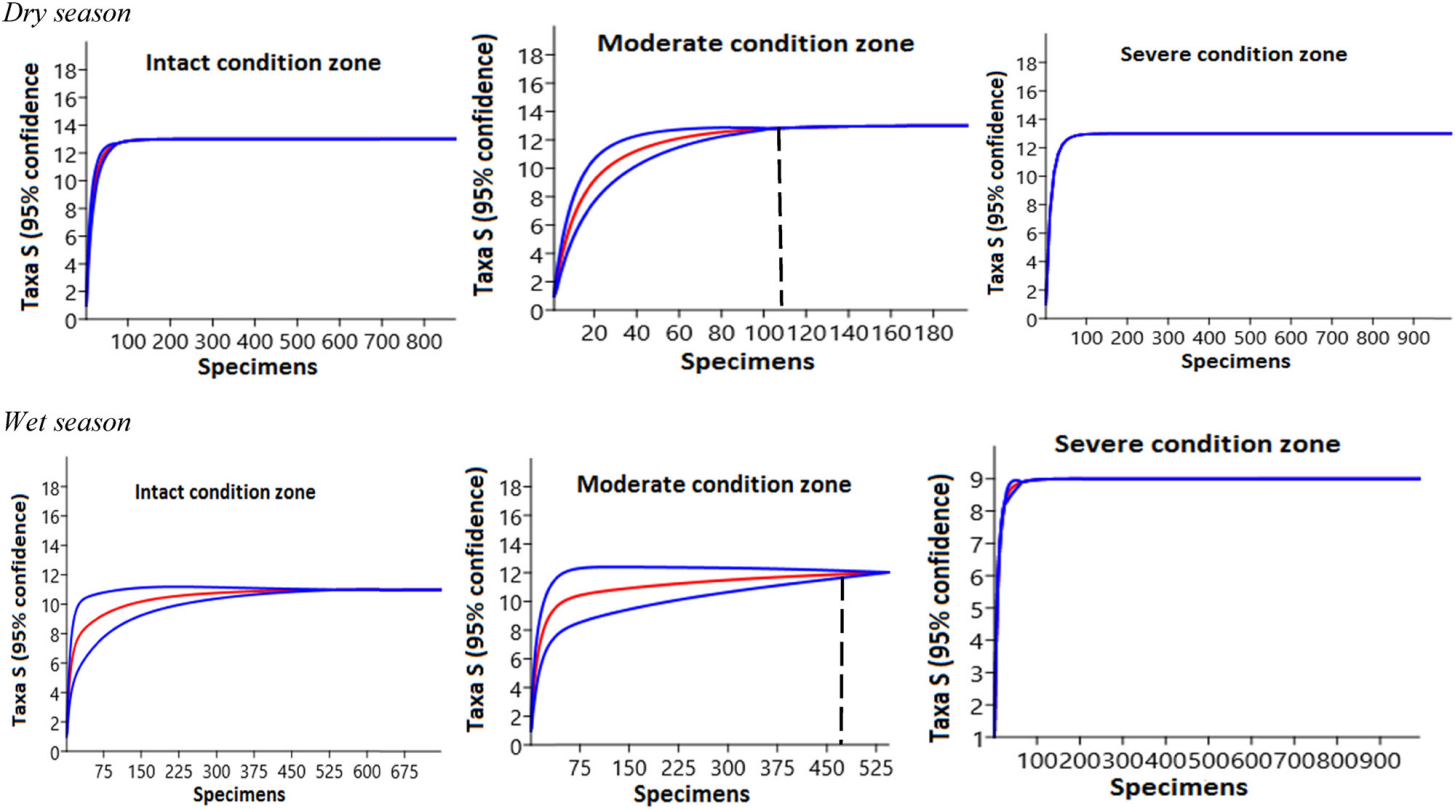
Standardized comparison of taxa richness for three individual-based rarefaction curves. Data are summary counts of invertebrate orders that were recorded from the three habitats in the dry and wet seasons. The red color lines are the rarefaction curves, calculated from equation (2) [44], with a 95% confidence interval in blue color. The dotted vertical lines illustrate a species taxa richness comparison standardized to 109 (dry season) and 423 (wet season) individuals, which was the least abundance registered in the moderately disturbed habitat. The smoothed average of these individual curves represents the statistical expectation of the species accumulation curve for that particular sample drawn on re-orderings, and the variability among the different orderings is reflected in the specific variance (conditional) in the number of species recorded for any given number of individuals.

**Figure 7:**
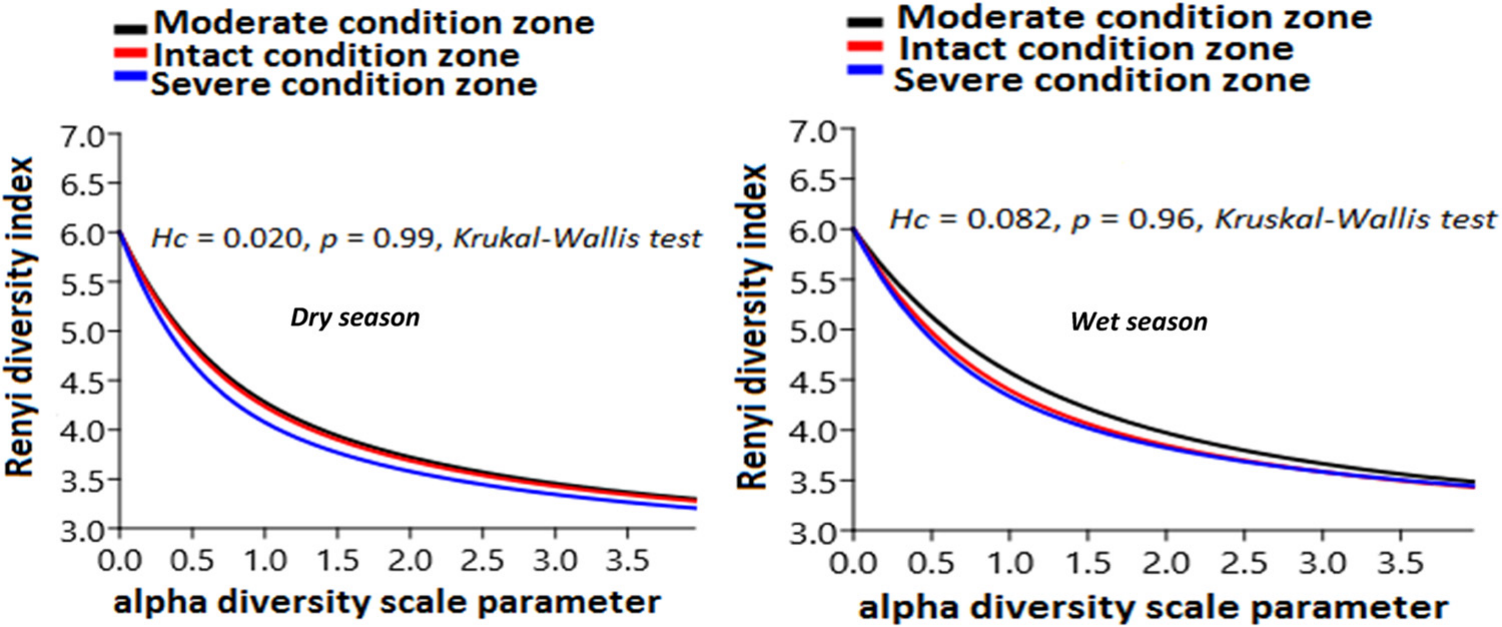
Renyi diversity ordering that compares invertebrate order evenness and richness among the habitats in the dry and wet seasons. Note that the shape of the curve for a site is an indication of its evenness profile. Thus, the shallower shape reflects high diversity and found on top of the curve, while the steeper shape curve indicates less diversity and found at the bottom. Notice that the moderately disturbed habitat (black color) is the shallowest curve and spatially evenly distributed, while the steeper curves were observed from the intact and severely disturbed habitats (red and blue colors, respectively).

Generally, seasonal insect order richness did not show any substantial variations among the three habitats (*t*-test = −1.084, *p* = 0.34), in spite of the mean insect order in the wet season (11.1 ± 2.0–2.6 ± 0.1) being slightly higher than the dry season (3.5 ± 0.4–2.3 ± 0.1) (Figure 6). Comparison among the three habitats showed that taxa richness in the intact (*n* = 13) and severely disturbed (*n* = 13) habitats were higher in the dry season than the moderately disturbed habitat (*n* = 12). However, in the wet season, taxa richness was highest in the intact habitat (*n* = 11). Variations in evenness distribution of insect order abundance were reflected in the shape of the Renyi diversity ordering profile (Figure 7). Thus, the habitat with the lowest number of individuals on the rank-abundance curve (i.e., shallower curve and higher evenness distribution) (Figure 5) was the most diverse on the Renyi diversity ordering profile and ranked highest along with alpha (*α*) scale values (Figure 7). Overall, the diversity of insect order did not differ in the dry (*H*_c_ = 0.020, *p* = 0.99, Kruskal–Wallis test) and wet (*H*_c_ = 0.082, *p* = 0.96, Kruskal–Wallis test) seasons.

From individual habitats, the moderate habitat zone (curve in black color) appeared the most diverse in the dry (*α* scale = 0.04, Renyi index (*r*) = 5.88 to *α* scale = 3.96, (*r*) = 3.30) and wet (*α* scale = 0.04, Renyi index (*r*) = 5.92 to *α* scale = 3.96, (*r*) = 3.49) seasons, as indicated in the shallowest curve (Figure 7). We observed in the dry season that insect order diversity in this habitat was closely similar to that of the intact habitat zone (curve in red color) (*α* scale = 0.04, (*r*) = 5.87 to *α* scale = 3.96, (*r*) = 3.28), as their profiles could barely be distinguished. The severe habitat zone was least diverse in the dry (*α* = 0.04, *r* = 5.85 to *α* = 3.96, *r* = 3.21) and wet (*α* = 0.04, *r* = 5.88 to *α* = 3.96, *r* = 3.44) seasons, as shown in the Renyi profile (lowest curve in blue color).

### 3.3 Environmental drivers influencing community assemblage of terrestrial invertebrates across the habitats in the Wewe river

**Figure 8:**
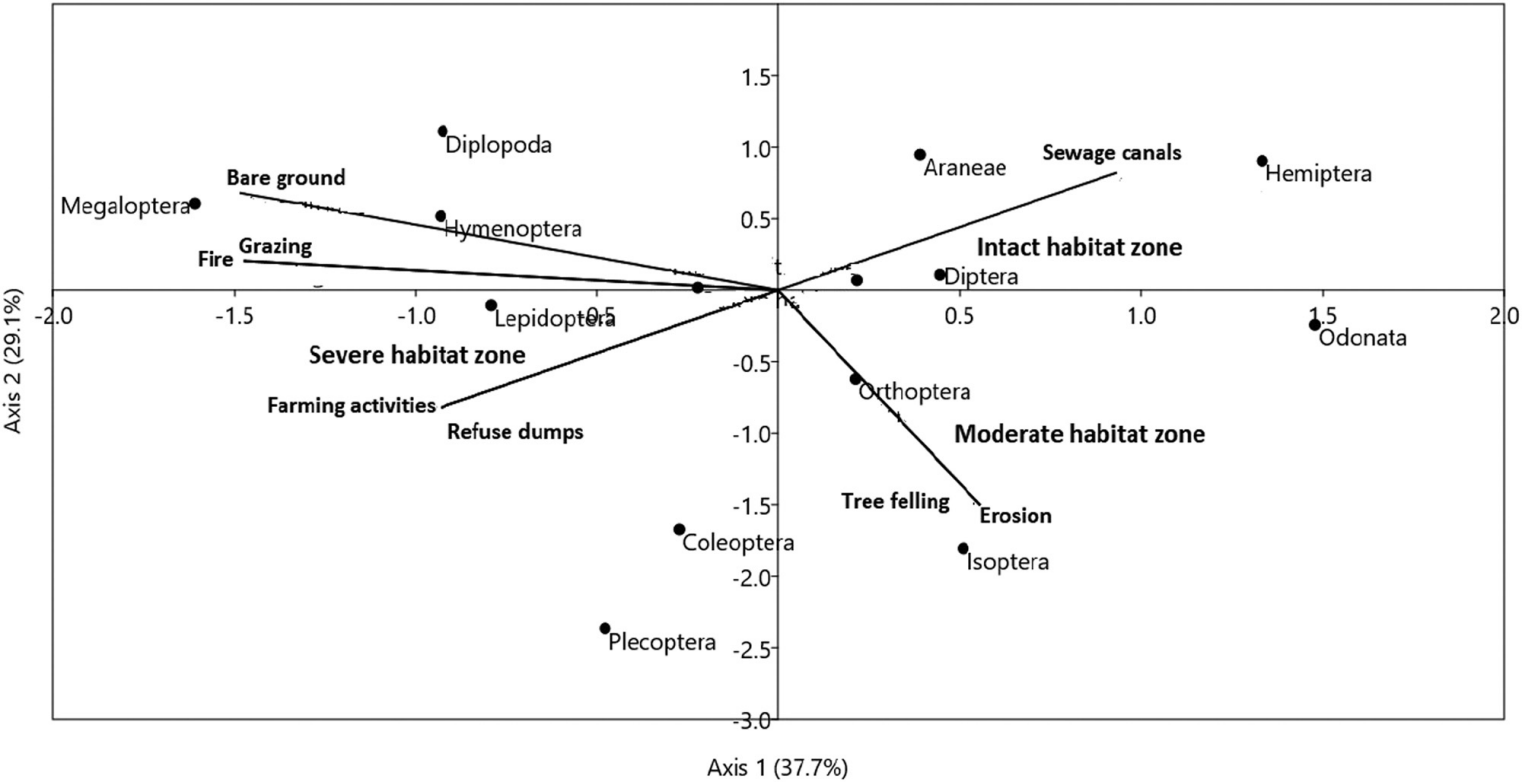
Canonical correspondence analysis (CCA) diagram showing the influence of environmental factors on invertebrate order assemblages. The first two axes (axis I = 37.71 and axis II = 29.05) explained 66.76% of the variance across the three habitats in the dry season. The arrows represent each of the environmental factors plotted pointing in the direction of maximum change of explanatory variables among the three habitats.

**Figure 9:**
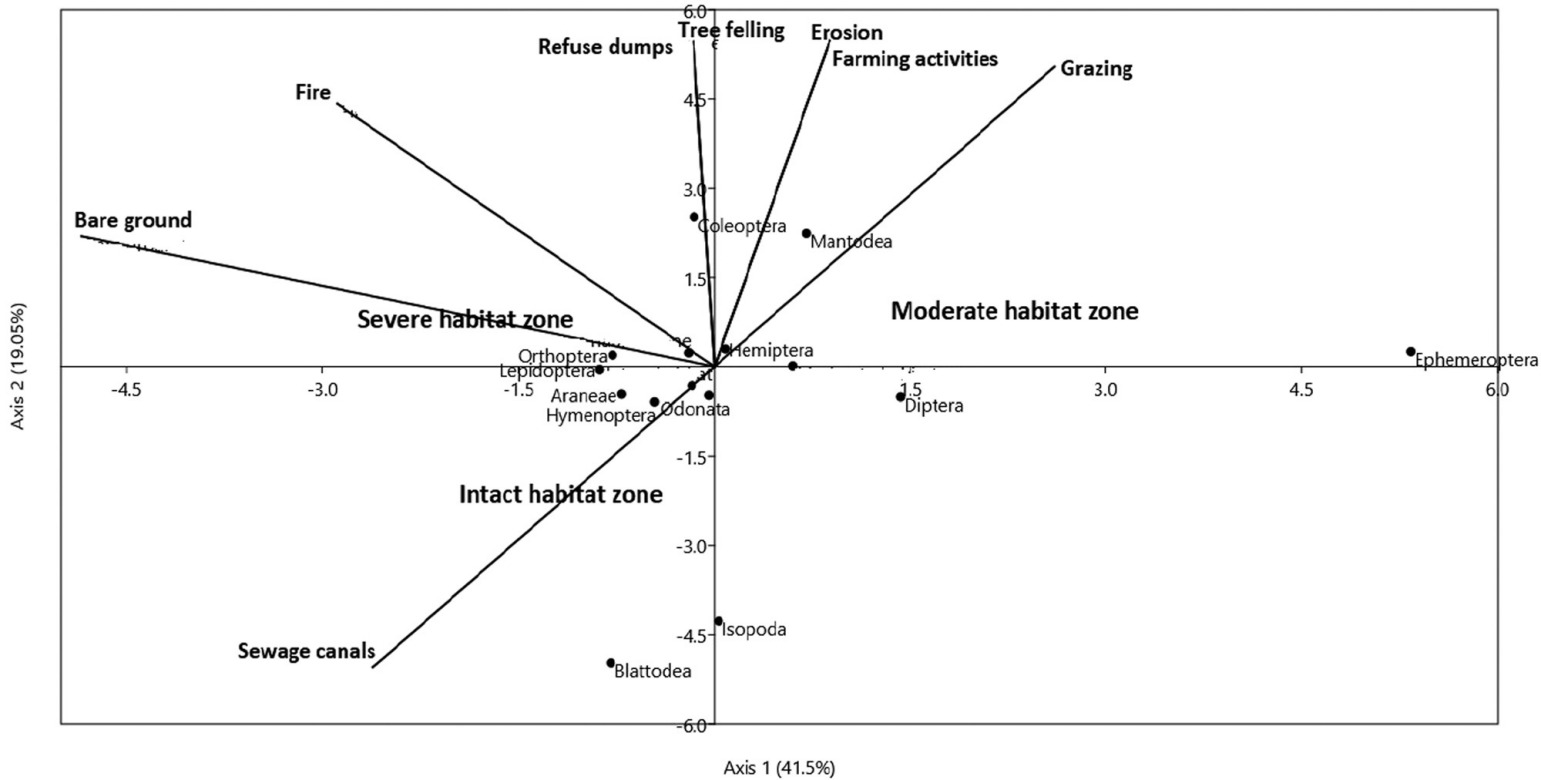
Canonical correspondence analysis (CCA) ordination diagram showing the influence of environmental factors on terrestrial invertebrate order assemblage. The first two axes (axis I = 41.5 and axis II = 19.05) explained 76.30% of the variance among the three habitats in the wet season. The arrows represent each of the environmental factors plotted pointing in the direction of maximum change of explanatory variables among the three habitats.

The summary of CCA ordination on the influence of environmental drivers on invertebrate assemblages is presented in Figures 8, 9 and Tables 3, 4. Environmental factors differed across the three habitats in the dry (*F*_2,21_ = 4.822, *P* < 0.02) and wet (*F*_2,21_ = 7.725, *P* < 0.003, one-way ANOVA test) seasons. Disturbances between the two seasons were also found to be significant (*F*_5,47_ = 5.272, *P* < 0.0007). Tukey *post hoc* test revealed intact × severely disturbed habitats in the dry season (*P* < 0.002), intact (wet season) × severely disturbed habitats (dry season) (*p* < 0.006), and severely disturbed habitat (wet season) × intact habitat (dry season) (*p* < 0.02) were the habitats that contributed to significant differences.

**Table 3:** Summary of canonical correspondence analysis (CCA) showing the levels of correlation between axes and environmental gradients, percentage variance of invertebrate order and order-environment relationships in both wet and dry seasons

Axis	Dry season		Wet season	
	I	II	I	II
Canonical eigenvalue	0.46	0.29	0.89	0.28
% variance explained	37.7	29.1	41.50	19.05
Correlations fire	0.85**	0.53*	0.41	−0.13
Farming	0.58*	−0.83**	0.78	0.21
Tree felling	−0.57*	−0.54	0.003	0.22
Erosion	−0.61*	−0.02	0.78	−0.20
Grazing	0.72**	0.53*	0.62	0.35
Bare ground	0.54*	0.26	−0.53	−0.41
Refuse dumps	0.44	−0.31	0.004	0.22
Sewage canals	−0.46	0.23	0.39	−0.17

∗ = (*p* < 0.05) and ** = (*p* < 0.01) following Monte Carlo permutation procedures.

CCA ordination for the dry season showed that fire (*r* = 0.85, *P* < 0.01), farming (*r* = 0.58, *P* < 0.05), tree felling (*r* = −0.57, *P* < 0.05), erosion (*r* = −0.61, *P* < 0.05), grazing (*r* = 0.72, *P* < 0.05), and bare ground (*r* = 0.54, *P* < 0.05) on axes I and II were the major drivers of invertebrate community structure in the dry season. The eigenvalues of the first two CCA axes (axis I = 0.46) and (axis II = 0.29) were significant (*P* < 0.01; 999 Monte Carlo permutation test). Insect orders such as Megaloptera, Hymenoptera, and Diplopoda, responded positively to the incidence of fire, bare ground, farming, and grazing activities in the severely disturbed habitat during the dry season. There was a strong intercorrelation among these environmental disturbances especially between fire and farming (*r*_s_ = 069, *P* < 0.05), erosion, and bare ground (*r*_s_ = 0.89, *P* < 0.01). The abundance of Hymenoptera for instance reflects their global least concern status (IUCN RedList) and tolerance to broad range disturbance scenarios (Figure 8 and Table 5). However, Plecoptera appeared to be negatively impacted by these threats, as their abundance was low, compared with the remaining insect orders in the same habitat.

In the moderately disturbed habitat, we found Isoptera (*n* = 12), Odonata (*n* = 13), and Orthoptera (*n* = 16) to have a narrow range distribution and less abundant in the moderately disturbed habitat whose substantial segment was severely eroded and characterized by tree felling. The low abundance of these insect orders is indicative of their sensitivity to habitat perturbation and this reflects in their categorization by IUCN RedList as critically threatened (CR), vulnerable (VU), and near threatened (NT), respectively. Endangered insect orders such as Araneae and Hemiptera were mostly dominant in the intact habitat zone where sewage canals were more widespread. In all, the first two axes (axis I = 37.7% and axis II = 29.1%) accounted for 66.8% of the variations in invertebrate assemblages in relation to eight environmental factors during the dry season (Figure 8 and Table 3).

In the wet season, farming activities (*r* = 0.78, *P* < 0.01), erosion (*r* = 0.74, *P* < 0.01), grazing (*r* = 0.62, *P* < 0.05), and bare ground (*r* = −0.53, *P* < 0.05) on axes I and II, were identified as the key determinants of invertebrate composition and abundance distribution (Figure 9 and Table 3). Total variability explained in invertebrate order assemblages was 60.55% (axis I = 41.5% and axis II = 19.05%) in relation to eight environmental factors (Figure 9 and Table 3). Othopthera showed a gradual rate of change in abundance, following the impact of fire and bare ground (*r*_s_ = 0.54, *P* < 0.05), fire and refuse dumps (*r*_s_ = 0.61, *P* < 0.05) in the severe habitat zone (Tables 3 and 4). Three insect orders namely Mantodea (*n* = 14), Coleoptera (*n* = 23), and Hemiptera (*n* = 29) in the moderately disturbed habitat, responded negatively to disturbances such as erosion and farming activities (*r*_s_ = 0.72, *P* < 0.01) and grazing and tree felling (*r*_s_ = 0.65, *P* < 0.05), as shown in their rank order abundance in the ordination diagram (Figure 9). Minimal disturbance in the intact habitat such as sewage spills through canals contributed to the high abundance of Araneae (*n* = 118), Odonata (*n* = 119), and Hymenoptera (*n* = 121). This habitat falls within the midstream segment of the Wewe river and serves as a transition zone between the moderately disturbed habitat (upstream) and severely disturbed habitat (downstream).

**Table 4:** Summary of Spearman rank (*r*_s_) correlation matrix between the environmental factors across the three habitats in the riparian zone

	Farming	Tree felling	Erosion	Grazing	Bare ground	Refuse dumps	Sewage canals
*Dry season*							
Fire	0.69*	0.81**	0.74**	0.36	0.56*	0.61*	0.67*
Farming		0.50*	0.65*	0.77**	0.63*	0.29	0.33
Tree felling			0.37	0.82**	0.86**	0.43	0.28
Erosion				0.86**	0.89**	0.38	0.51*
Grazing					0.87**	0.45	0.85*
Bare ground						0.65*	0.25
Refuse dumps							0.34
Sewage canals							

*Wet season*							
Fire	0.86**	0.67*	0.72*	0.85**	0.54*	0.66*	0.67*
Farming		0.33	0.35	0.65*	0.88**	0.31	0.68*
Tree felling			0.32	0.69*	0.75**	0.42	0.61*
Erosion				0.52*	0.81**	0.35	0.52*
Grazing					0.56*	0.43	0.40
Bare ground						0.27	0.53*
Refuse dumps							0.63*
Sewage canals							

Significance of *R*-values: *p < 0.05; **p = 0.01.

**Table 5:** IUCN “Red List” conservation status categorizations for terrestrial invertebrate orders sampled along with the three segments in the riparian zone of the Wewe river. LC = least concern; NT = near threatened; CR = critical endangered; EN = endangered; VU = vulnerable (IUCN, 2011)

Order	Number of Individual in each habitat type			IUCN conservation status
	Moderate disturbance	Intact zone	Severe disturbance	
Araneae	38	238	225	Endangered
Blattodea	0	9	0	Endangered
Coleoptera	46	107	229	Endangered
Diptera	159	214	178	Least concern
Diplopoda	0	62	91	Vulnerable
Ephemeroptera	40	42	95	Endangered
Hemiptera	36	303	232	Endangered
Hymenoptera	51	188	219	Least concern
Isopoda	0	13	0	Least concern
Isoptera	16	56	51	Least concern
Lepidoptera	21	149	184	Near threatened
Mantodea	14	8	33	Near threatened
Odonata	76	228	176	Vulnerable
Orthoptera	24	148	167	Near threatened
Plecoptera	9	21	32	Vulnerable
Trichoptera	51	82	445	Least concern

## 4 Discussion

Disturbances around the catchment of riverine systems in urban centers have often led to transformation or loss of riparian vegetation [5,6,9,58] which largely serves as a refugium for invertebrates [7] and consequently affect invertebrate assemblages [9]. In this study, we found disturbances such as fire, grazing, farming activities, erosion, bare ground, tree felling, and refuse dumps as the major drivers of invertebrate community structure and distribution along the riparian zone of the Wewe river. Some invertebrates of conservation concern namely Isoptera (CR), Odonata (VU), Plecoptera (VU), and Orthoptera (NT) were less abundant, with narrow range distribution in the moderately disturbed habitat. The low abundance of these orders in the moderately disturbed habitat suggests their sensitivity to perturbation, which was characterized by erosion and widespread tree felling. Other studies have found the population decline of invertebrates and the altering of their composition to be linked largely to human activities such as farming activities, logging pasturing, erosion, burning, and urbanization along rivers and streams [13,58,59]. This rapid decline of insect pollinators and a shift in their community ranges could soon translate into less frequent flower visitation and gradual reduction of seed and fruit production [12]. This phenomenon could get worse, given that similar anthropogenic disturbances have led to a 45% reduction in global terrestrial invertebrate populations in the current anthropocene epoch, with the likelihood of impairing ecosystem functioning and human well-being [11]. The broad range distribution of Hymenoptera across the three habitats was probably because over time and their morphology and physiology have been modified to adapt to the changing environment brought about by disturbances. A Nationwide study of invertebrate assemblages in Korean wetlands showed that invertebrates encountered in various habitat types may have grown to tolerate a wide range of ecological conditions [60]. Thus, it can be inferred from this study that the abundance and widespread distribution of Hymenoptera across different habitat zones may well explain why they are classified as being of globally Least Concern Status by IUCN “RedList”.

The dominance of Araneae, Odonata (i.e., mostly zygopterans), and Hymenoptera in the intact habitat compared with the remaining two habitats could be due to the minimal disturbances therein and the diverse vegetation, typical of gallery forest. This segment of the riparian zone falls within the midstream of the Wewe river and serves as a transition riparian zone between the severely disturbed habitat (downstream) and the moderately disturbed habitat (upstream). Thus, specialist insect orders such as Odonata considered this habitat zone as a safe haven to colonize, while other generalist orders also considered this habitat as refugia for migration during extreme disturbances well beyond their threshold tolerance, from either the severe or the moderately disturbed habitat. Support for specialists and generalists in the intact habitat could greatly contribute to increased taxa richness of the riparian zone. Ramey and Richardson [7] listed five characteristics of riparian zones that may support specialist riparian invertebrates to include low disturbance, elevated nutrient and water availability, increased vegetation and microhabitat diversity, strong microclimate gradients, and unique food resources.

The complete absence of Plecoptera (stoneflies) and Isoptera (termites) in the three habitats during the wet season may be attributed to the increased levels of environmental disturbances, such as grazing and farming activities. It may be the case that at some stage in their life cycle, these insect orders require favorable environmental conditions to thrive. Hence, conditions outside their tolerance range could offset their survival. For instance, Isopterans are active soil invertebrates with most of their life activities occurring below the soil surface, because of their Saproxylic nature (defined here as “insects that depend on dead or dying wood of moribound or dead trees during some part of their lifetime, or upon wood-inhabiting fungi”) [61]. Thus, in the dry season when soils become drier and compacted (e.g., due to incidence of fire), Isopterans are restricted from burrowing activities in search of food. This compels them to move around the ground surface in search of food and water. Whereas in the wet season, the absence or reduction in burning and increased soil moisture makes it possible for the Isopterans to burrow below the soil surface.

## 5 Conclusions

Overall, invertebrate assemblages varied with seasons. The severely disturbed and intact habitats registered the largest number of individuals and richness, whereas the moderately disturbed habitat was the most diverse. Hymenoptera was the only invertebrate order that was found to have a broad range of distribution and tolerance to disturbances in both seasons. Some invertebrates such as Plecoptera (stoneflies) and Isoptera (termites) were largely influenced by seasonality, as they only occurred in the wet season. Environmental disturbances, namely, fire, farming activities, erosion, bare ground, and refuse dumps, contributed substantially in influencing invertebrate composition, abundance, and distribution patterns across the three habitats. These disturbances led to the narrow range distribution and lower abundance of Isoptera, Odonata, and Orthoptera, especially in the moderately disturbed habitat. The low abundance of these insect orders is indicative of their sensitivity to habitat perturbation and this reflects in their categorization by IUCN RedList as Endangered (EN), critical (CR), vulnerable (VU), and near threatened (NT), respectively. Given the level of threats in the riparian zone and its direct effect on the overall functioning status of the Wewe river, it is recommended that the following conservation measures be considered to revert the following threats: (a) all future structural development should be sited as far away from the river as possible, to help reduce its impact in the riparian zone; (b) tree planting should be undertaken as a means of restoring the moderate and severely disturbed habitats, which were characterized by widespread tree felling, erosion, bare ground, and farming activities; (c) farming activities within the riparian zone should be banned and farmers re-located in places further away from the riparian zones. Additionally, warning signposts should be placed at vantage points along the riparian zone, with inscriptions on penalties when culprits are caught in the act of burning, farming, or refuse disposal. This warning should be complemented by regular monitoring of the catchment of the riparian zone; (d) all sewage spillways should be diverted away from the riparian zone, to prevent pollution and algal bloom.
